# Multisystem compensations and consequences in spastic quadriplegic cerebral palsy children

**DOI:** 10.3389/fneur.2022.1076316

**Published:** 2023-01-09

**Authors:** Luh Karunia Wahyuni

**Affiliations:** Physical Medicine and Rehabilitation Department, Dr. Cipto Mangunkusumo Hospital - Faculty of Medicine Universitas Indonesia, Jakarta, Indonesia

**Keywords:** spastic quadriplegic cerebral palsy children, atypical movement pattern, compensatory strategy, multisystem consequences, total body extension

## Abstract

Spastic quadriplegic cerebral palsy (CP) is a permanent neuromuscular disorder causing limitation on all four limbs following a lesion on the developing brain. Most children with spastic quadriplegic CP are identified to be Gross Motor Function Classification System (GMFCS) level V, thus they have more comorbidities compared to other types at lower levels. Spastic quadriplegic CP is characterized by weak and inactive postural muscles of the neck and trunk, hence, they will undergo a total body extension as a compensatory mechanism leading to an atypical movement pattern, that give rise to multisystem consequences that reduce their quality of life. The relationship between atypical movement patterns, compensatory strategies, and multisystem consequences have not yet been explored. In fact, these multisystem consequences aggravate their condition and make movement much more atypical, forming a vicious cycle. This review aimed to provide a summary and highlight the mechanism of atypical movement pattern, multisystem compensations, and consequences in spastic quadriplegic CP children. It is true that central nervous system (CNS) lesion in CP is non-progressive, however the multisystem consequences may impair overall function over time. An understanding of how compensatory strategy and multisystem consequences in spastic quadriplegic CP offers the opportunity to intervene as early as possible to improve their quality of life.

## 1. Introduction

Cerebral palsy (CP) is a group of permanent disorders that affect the development of movement and posture, causing activity limitations that are attributed to non-progressive disturbances of the developing fetal or brain (1). CP is the most frequent cause of motor disability in children and adolescents that are often accompanied by disturbances in cognition, perception, sensation, communication, behavior, and/or a seizure disorder (2, 3).

As injury to the developing brain occurs, due to numerous causes in different clinical manifestations and severity, CP has been described in various headings based on: the type of movement disorder, area of the body involved, and level of damage (4). Traditionally, two groups of movement disorders are described as: (1) pyramidal (spastic) and (2) extrapyramidal (ataxic, athetoid, and/or dystonic). However, it is recognized that CP children often have co-existing pyramidal and extrapyramidal signs and can be described as mixed, or classified according to the movement disorder considered predominant (5). These motor disturbances exist and may change during the early years of life with spastic CP being the most common type, accounting for up to 75% of the cases (6). Based on the area of presentation, spastic CP can be classified into involvement of whichever side of the body is affected: quadriplegic, hemiplegic, diplegic, or monoplegic. In quadriplegic CP, all four limbs are affected, causing limited voluntary movements of all extremities, pseudobulbar signs, food aspiration, dysphagia, optic atrophy, seizures, and severe intellectual abnormality (4).

Functional classification is necessary to recognize the differences in stages of cerebral palsy for predicting the functioning of the affected limbs and the treatment's outcome. The severity of motor impairment is evaluated by the Gross Motor Function Classification System (GMFCS) as a widely used standard measure of motor function in CP developed by Palisano et al. (7) GMFCS describes movement ability of children with CP into one of five ordinal levels (I–V). Children at level V have difficulty controlling their head and trunk posture in most positions, and achieving any voluntary control of movement, hence they are dependent on all settings and have limitations to maintain antigravity posture (8).

A study in the United States claimed that the highest proportion of children with CP are classified as GMFCS levels I–II (20%–35%) (9). This is contradictory to what has been declared in Indonesia, as Nur, Handryastuti, and Poesponegoro (10) had stated, the occurrence of CP classified as GMFCS V and spastic quadriplegic is quite high (33.7 and 41.2% of 80 CP cases respectively). CP severity in children and adolescents are found to be less severe in developed countries compared to developing countries (11, 12). This discrepancy may be caused by the underdevelopment of early detection, diagnostic, and therapeutic procedures of CP in developing countries. Most patients with quadriplegic spastic CP are identified to be GMFCS level V, thus these patients have poor ability to move without the help of others, and have more comorbidities compared to other types at lower levels (10).

Previous studies have shown that the lesions in CP, especially in spastic quadriplegic children, often cause damage to more than one system and resulting in impairments that influence movement control and other functions. Atypical movement patterns are those that are immediately and directly a result of the lesion. Compensations and consequences develop in systems or organs over time because of the effects of one or more primary impairments and may become just as debilitating as those atypical movement patterns (13).

Understanding the development of multisystem compensations and consequences is extremely important. Most healthcare professionals, particularly in Indonesia, who are involved in treating CP children have not had much opportunity to observe and think about changes that occur over time. It is true that central nervous system (CNS) lesion in CP is non-progressive, however the multisystem consequences may impair overall function over time (4).

Healthcare professionals may have a stronger influence on the course of development of these multisystem compensations and consequences than on the primary impairments. An understanding of how multisystem compensations and consequences develop offers the opportunity to intervene as much as possible before they begin. This review aimed to provide a summary and highlight the mechanism of atypical movement pattern, multisystem compensations and consequences in spastic quadriplegic CP children.

## 2. Atypical movement pattern and compensatory strategy

**Figure 1:**
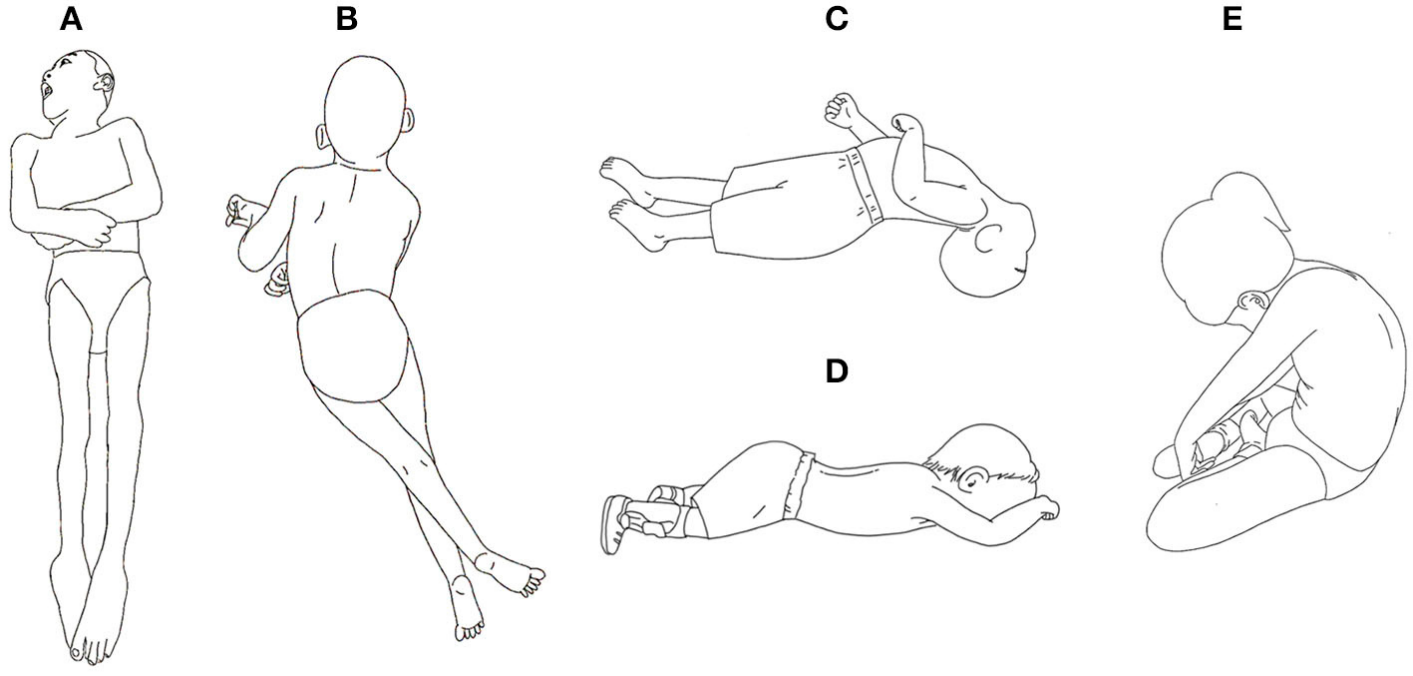
Typical posture of a child with spastic quadriplegic CP: **(A)** supine; **(B)** prone; **(C)** lateral view of supine; **(D)** lateral view of prone; **(E)** sitting.

The child with CP spastic quadriplegic cannot bring the head to the midline and hold it there, and cannot tuck the chin (capital flexion) in a supine position because the postural muscles of the neck and trunk are weak and inactive. The child may use shoulder (scapular) elevation, adduction, and internal rotation as a compensatory strategy to stabilize the head, therefore preventing typical head/neck movements. The child has difficulty with control of the scapulae on the thorax, and is unable to use active extension throughout the thoracic spine or forearm and extended arm weight bearing. If the child does not bear weight on the arms, proprioceptive input is limited, and cannot progress to the next steps of extended arm weight bearing and forearm weight shifting. The child who cannot shift weight in a typical way often tries to shift weight with head (cervical) extension, scapular adduction, and total body extension (14, 15). By trying harder, the body achieves more extension and maintains that extension because this is what the central nervous system is capable of (16). The child may use strong asymmetrical head/neck extension to initiate movements in all positions: supine, prone, and sitting (Figure 1).

Total body extension hinders the regulation of pressure within the thoracic and abdominal cavities by poor postural control, unbalanced gravitational influence, and restricted diaphragmatic movement. Poor postural control over the head, neck, trunk, and abdominal muscles cause an elevated and immobile ribcage, which limits its expansion. This condition forces the diaphragm to work harder to regulate intrathoracic and intra-abdominal pressure which leads to fatigue (17). Spastic quadriplegic CP children typically spend more time being in a supine position, which leads to an unbalanced gravitational influence and undesirable changes in the thorax that impairs their ventilation. Furthermore, diaphragmatic movement in spastic quadriplegic CP is restrained due to lesions in the primary cortex and corticospinal tract, causing a decreased muscle strength. All these impairments are causing the loss of the ability to generate, regulate, and/or maintain appropriate internal pressure in both thoracic and abdominal cavities. As a result, the mechanics of breathing may be impaired as well as induce multisystem consequences: limited oromotor function, drooling, swallowing difficulty, gastroesophageal reflux, constipation, sleep disorder, and pain (18). With exclusive use of cervical extension, shoulder elevation and internal rotation, the pectorals shorten, as do the latissimus dorsi. With this proximal posturing, the forearm tends to pronate with an elbow, wrist, and fingers flexion and ulnar deviation (16).

The child may show a variable posture in the trunk when in different positions. The child may show lumbar extension in prone and supine (Figures 1A–D), but show flexion throughout the lumbar spine when sitting (Figure 1E). The deeper postural extensors such as the multifidus, rotators and abdominal muscles are weak and inactive, often sustaining muscle activity in muscles that are superficial across more than one joint, and or were normally short at birth. The erector spinae are found in many joints and are superficial muscles, so they are easier to recruit, resulting in lumbar extension more easily than thoracic extension. The easiest synergy for the child is lumbar extension with hip flexion (16).

Rectus abdominis is the abdominal muscle that a child with quadriplegia is able to recruit in the abdominal group, bringing the muscles' origin closer to its insertion, especially when sitting. The child uses active holding in trunk flexion with the pectorals and rectus abdominis. This also is aided by the strong contraction of the hip flexors and hamstrings, which pull the pelvis into a posterior tilt (16). Consequently, the child sits on his sacrum rather than the ischial tuberosities. Subsequently, tightness of the hip extensors causes a posterior pelvic tilt, lumbar and thoracic flexion. To reduce the pull of the tight hip extensors, the child's knees flex and put the hamstrings on a slack. If the child stays in this flexed position for a long time, he begins to fix the rectus abdominis muscle.

Total body extension prompts the child to not be able to learn dissociated movements. As the legs do not move with alternating movements, there is a lack of lower extremity kicking in and out of flexion in supine (14). This condition is also accompanied by inactive abdominal muscles. Because the child does not practice active flexion, abduction, or external rotation of the lower extremities, the extensor and adductor muscles are never balanced or elongated. Long-term unstimulated muscles are not able to resist the forces of gravity resulting in muscular weakness and atrophy which condemn their development and postural stability. Impaired posture leads to higher risks of musculoskeletal disorders (hip subluxation/dislocation, scoliosis, and joint contracture) (16, 18).

Strong lower extremity extension in prone and supine causes the lumbar spine to extend and further extends the rest of the spine. The compensations of the child with a high tone and marked extension are usually not a result of his efforts, but are a result of the positions in which he is placed. Because of the shortened muscles, the child does not have typical joint mobility. Therefore, the child obtains mobility for the various positions in which he is placed (especially sitting) from the body's points of least resistance (16).

Ironically, the child who is very extended often becomes very flexed as the child gets older because of the compensations that occur in sitting. The child develops atypical spinal flexion, posterior pelvic tilting, and hip and knee flexion. The flexor muscles subsequently become tight. In the older child, excessive flexion seems to be the major problem. However, the excessive flexion is a compensation (16).

**Figure 2:**
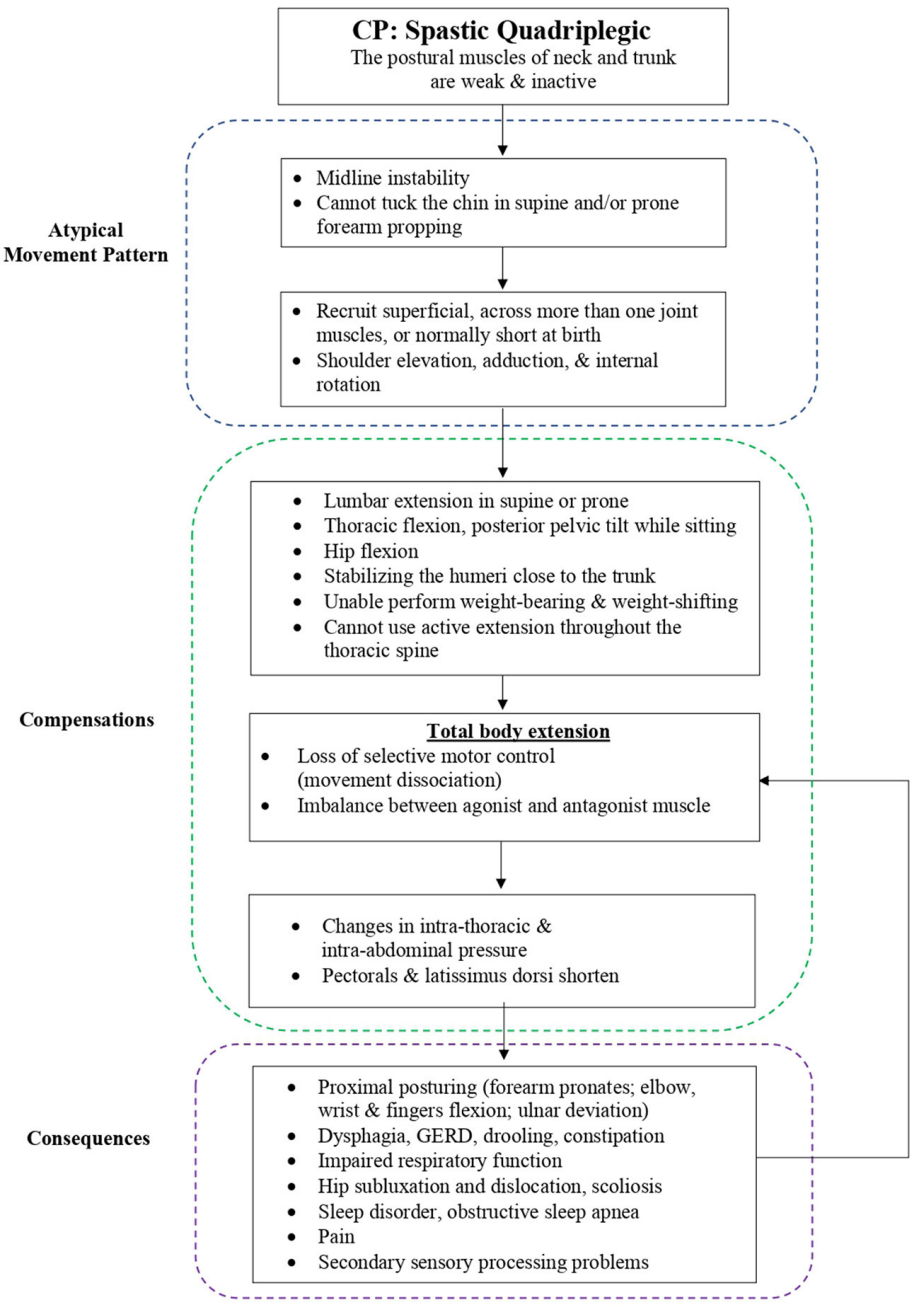
Mechanism of atypical movement pattern, multisystem compensations, and consequences of spastic quadriplegic cerebral palsy in children.

Mechanism of atypical movement pattern, multisystem compensations, and consequences of spastic quadriplegic CP children is presented in (Figure 2).

## 3. Multisystem consequences of atypical movement pattern

Spastic quadriplegic CP is a heterogeneous condition with a wide range of consequences that rise from an atypical movement pattern. Total body extension is the core problem that gives rise to these consequences ranging in a multisystem function. Our aim is to highlight these consequences as a result of total body extension.

### 3.1. Dysphagia

The presence of motor dysfunction in spastic quadriplegic CP affects their ability to feed, several causes include inadequate postural control, involuntary movements, immobility, self-feed inability, and seating difficulties. To achieve functional and safe feeding, a stable posture is needed as children who are insecure may experience fear and anxiety and further complicate the feeding process. Limited oromotor function in CP increases susceptibility to aspiration because of an abnormal head posture created from the close interdependency between cranio-cervical posture and pharyngeal airway stability. Airway structure is dependent on neck position, flexing the neck will reduce the airway but enlarges the vallecula space due to the tongue falling forward, causing the epiglottis to fall backward and overhangs above the airway. Epiglottis in this condition serves as an airway's mechanism of protection, however, the enlarged vallecular space increases the accumulation of residue.

Numerous studies have shown that higher levels of motor impairment (GMFCS IV–V) were more likely to be associated with worse dysphagia symptoms and more frequent occurrences of malnutrition (19–21). It was also stated that there was a five-fold increase in GMFCS IV and a 15-fold increase in GMFCS V of having swallowing difficulties (22). The prevalence of dysphagia in CP ranges from 21% in a group of 1,357 children (22) to 99% in a group of 166 severely generalized CP with intellectual disability (20).

Dysphagia in CP is described as poor tongue function impacting on bolus transport, delayed swallow initiation, and reduced pharyngeal motility (23). Neurological lesion in CP causes neuromuscular dysfunction which may have a direct or indirect effect on oral motor disorders, pharyngoesophageal dyskinesia, and/or intestinal dysmotility (21). Furthermore, physical abnormalities including posture, tone, and movement disorders in spastic quadriplegic CP children can result in abnormal motor development that affects the feeding process itself. GMFCS V children had feeding posture compromised, which can affect their swallow by promoting poor alignment or reducing the stability for controlled oral movements. Tone abnormalities of the neck and trunk will also cause difficulty in swallowing (24). Dysphagia may hinder the safety and efficiency of feeding, as it is often associated with recurrent aspiration, prolonged feeding times, delayed progression of oral feeding skills, and also inducing stress during mealtimes for the child and the caregiver. As a result, this condition may lead to aspiration pneumonia, inadequate nutritional status, and further impaired child's growth (25).

The primary goals of dysphagia interventions are to control treatable causes and avoid or minimize the impact of swallowing dysfunction. Clinicians first should consider the implementation of the safest, least invasive, and most functional management options. Modifications in the texture of food or liquids, the use of utensils, the position or scheduling of feeding, tactile stimulation, and activities that focus on swallowing musculature are essential. Short or long-term supplemental tube feedings may be considered for children who have oropharyngeal dysphagia associated with respiratory compromise or the inability to meet nutrition needs safely (26).

### 3.2. Gastroesophageal reflux disease

Gastroesophageal reflux disease (GERD) is defined as the pathologic sequelae of retrograde gastric contents movement into the esophagus as it leads to symptoms or complications such as dysphagia, dental erosions, poor weight gain, and vomiting (27, 28). Children with spastic quadriplegic GMFCS V cerebral palsy (CP) are at an increased risk for GERD due to a number of contributing factors such as chronic supine position (immobilization), scoliosis which may displace the stomach and stretch the lower esophageal sphincter together with a spastic body state will increase intraabdominal pressure, therefore promote GERD, delayed gastric emptying, inadequate oral intake, and abnormal autonomic control of gastrointestinal motility as it leads to an impaired clearance to refluxate (27). Although literature discussing GERD and CP is scarce, the incidence of GERD in children with CP has been reported to vary from 15% to 91% by different studies (28, 29).

CP spastic quadriplegic with GERD may present with feeding difficulties (refusal and/or intolerance); recurrent vomiting, nausea, heartburn, and pain in the epigastric area; the presence of dysphagia and/or odynophagia; and GERD may exacerbate and worsen respiratory diseases, either by aspirations of refluxate leading to inflammatory changes in the respiratory tract or by ineffective reflexive responses of the airway to refluxed material such as coughing, laryngospasm, and apnea (27). Oromotor dysfunction and abnormalities of pharyngeal coordination can sometimes mask or be confused with GERD (28).

Medications used frequently in the treatment of spasticity and seizures associated with CP patients have side effects that can cause delayed gastrointestinal motility, so it is best to consider the side effects as an exacerbating factor and withdrawal symptoms (use of baclofen, opioids, benzodiazepines, tricyclic antidepressants) that may result in GERD (27, 29, 30).

Various treatment strategies for the management of GERD include lifestyle modification, medications and surgical options. As feeding and swallowing dysfunction is very common in CP children, some may require feeds *via* a feeding tube. Adjustments to the feeding volume, frequency, and length of administration can be made to decrease the gastric volume and allow adequate time for gastric clearance. Besides, ensuring an upright position especially during meals can help decreasing the frequency of reflux episodes. The different treatment approaches available may have variable degrees of effectiveness in each case, but timely management can prevent serious complications from GERD (27, 29).

### 3.3. Drooling

Drooling, or sialorrhea is defined as an uncontrollable and continuous secretion of saliva resulting in the unintentional loss of saliva in the oral cavity (anterior drooling) or the hypopharynx (posterior drooling). Drooling is considered to be part of normal development in children, however, it is declared as an abnormality after the age of 4 years (31, 32). It is estimated that 10%–37% of children with CP experience drooling, a study in 2015 by Edvinsson and Lundqvist (33) stated that drooling occurrence is more likely to be found in worse gross motor functions in CP children [95% in GMFCS V compared to; 13% (I), 36% (II), 20% (III), 61% (IV)] and Fairhurst (34) stated a higher prevalence in spastic quadriplegic type. Drooling in CP is not necessarily related to excessive saliva production, however, it is closely linked to the dysfunction of oromotor control, which is often characterized by reduced lip closure and frequency of spontaneous swallowing, difficulty in formatting food bolus, and suction pressure, lack of voluntary control of tongue and mandible, and decreased intraoral sensitivity (35, 36). Furthermore, poor head and neck control ensues a forward-leaning posture in CP and may also decrease oromotor and facial sensation (31).

Drooling may cause several issues in a child's health and development. Anterior drooling in excessive amounts may cause chapped skin, dental occlusion, poor oral hygiene, dehydration, chewing disorders, loss of self-confidence, and social isolation. Meanwhile, posterior drooling leads to serious health problems: shortness of breath, coughing, choking, vomiting, and aspiration which can cause life-threatening pneumonia (31, 32). Other medical conditions in CP such as GERD, dental infections, and anti-epileptic drug use such as clonazepam can cause an acute increase in saliva production (hypersialia) (32, 36).

Management of drooling in CP involves managing its potential exacerbating factors. Maintenance of good oral health and avoidance of sweet or acidic foods which stimulate saliva production are very important to prevent drooling. Specific tongue and mouth exercises may also help children to develop saliva control. If drooling has coincided with a change of medication, it may be necessary to explore this and consider alternative options (37).

### 3.4. Impaired respiratory functions

Respiratory consequences in CP spastic quadriplegic children is a complex multifactorial disease process and is the most common cause of morbidity and mortality in CP children (38). Approximately 40% of children with spastic CP have impaired respiratory functions, which include symptoms such as dyspnea, cough, snoring, wheezing, crackles, asthma episodes, or regurgitating during, or after meals (39, 40). It is also reported that spastic quadriplegic CP with GMFCS V has much higher rates of these symptoms compared to other levels (40, 41).

Previous studies have shown that impaired respiratory function in spastic quadriplegic CP children is associated with respiratory muscle paralysis, inefficient biomechanics of breathing structures, limited chest expansion, and immobility (41, 42). Diaphragmatic movement that aids normal respiration is restrained in non-ambulatory spastic quadriplegic CP due to damage to the primary cortex and corticospinal tract, where a decrease in sarcomeres results in decreased muscle strength (16). Oropharyngeal dysphagia and gastroesophageal reflux may promote aspiration (38). Scoliosis is also a risk factor, as a deformed thoracic cage causes reduced lung volume and lung compliance, a reduced force of the respiratory muscles, and increased stiffness of the chest wall (40, 43). Furthermore, individuals with more severe gross motor function impairment are known to be almost always in a recumbent position, and only sit up to take meals and immediately get back to the lying position (44). All these factors increase the risk for respiratory complications such as recurrent aspiration pneumonia, atelectasis; bronchiectasis, chronic obstructive and restrictive lung diseases in CP children (41).

Management of impaired respiratory functions is treating the respective underlying causes. Surgery for scoliosis should be considered when a curve exceeds 50° or functional sitting deteriorates. Proton pump inhibitors and lifestyle modifications (thickened feeds, avoiding acidic or spicy foods, sitting upright during eating) may help to reduce the risk of aspiration. In CP children with persistent respiratory symptoms, physiotherapists educate families/carers in positioning to optimize lung function, maintain chest wall mobility, and teach home airway clearance regimes if necessary (38).

### 3.5. Constipation

Constipation or dysmotility of the gut is the most common gastrointestinal symptom that negatively affects the quality of life of non-ambulatory children with cerebral palsy (GMFCS IV–V) (45). Although no sources had specifically defined constipated children with limited ambulatory functions, several studies had it defined as similar to constipation in typical developing children or adults with intellectual limitations, where scybalous, pebble-like, hard stools are present in more than 25% of defecations and defecation frequency is less than three times a week, when large stools are palpable on abdominal examination, or when laxatives or manual disimpaction are used (46, 47). Constipation in CP incidence rate ranges from 25–75% from multiple studies because of the differences between each source's definition of constipation, diagnostic method, and subjects used (45–47).

Constipation in CP is hypothesized to be caused by various factors including poor gastrointestinal motility, diet, medications, pelvic muscular instability and increased intra-abdominal pressure (45, 47–49). Park et al. (48) studied colon transit time to diagnose constipation in children with CP, where the left colon was concluded to have the longest transit time. Tube-fed children are likely to lack dietary fibers which results in harder stools and further complicated defecation disorders. Drugs that are commonly used in cerebral palsy such as botulinum toxin type A (BoMT-A), anticonvulsants, and anticholinergic drugs may further cause colonic motility impairment (50).

Several managements for treating constipation in children with CP include adequate fluid intakes, use of fiber diet, biofeedback, use of oral laxatives, and rectal stimulants. Furthermore, non-pharmacology treatment, particularly physical therapy aims to improve spasticity, range of motion (ROM), and mobility contribute in relieving constipation in children with CP (51).

### 3.6. Hip subluxation and dislocation

Hip subluxation is a common progressive condition found in CP children, and is attributed to spasticity and contracture of the hip adductors and flexors as well as the medial hamstrings, silently resulting in dislocation if left untreated (52). Larnert et al. (53) stated that children at GMFCS V had a 2.5–3 times higher risk of developing hip subluxation compared with children at GMFCS III–IV, with the highest annual incidence by age 2–3 years and 7–8 years of age. The incidence of dislocation increases with age, but the tendency of subluxation is more pronounced in earlier ages with motor impairment, thus CP patients categorized as GMFCS V are more likely to develop hip subluxation at earlier stages in life (53).

Spasticity in CP stiffens the muscles, resulting in a fixed myostatic contracture of the hip adductor and flexor muscles. These muscles pull the proximal femur to adduction, flexion, and internal rotation, constructively guiding the femoral head away from the hip joint resulting in subluxation. Another factor that can give rise to subluxation is the abductor muscle weakness that delays the weight-bearing ability of a patient through progressive acetabular dysplasia and changes in proximal femoral geometry: increased femoral neck anteversion, coxa valga, and horizontal growth plate (54, 55). Children with GMFCS V lack head control, have no sitting balance, have no independent mobility, and usually have severe spastic dystonia in a quadriplegic pattern (52).

Hip subluxation may induce muscle imbalance, osseous deformity, including increased femoral anteversion and acetabular dysplasia, which further increases the risk of hip instability. Unilateral hip dislocation is sometimes associated with the development of pelvic obliquity and scoliosis. Dislocation causes poor quality of life and pain in children due to swelling of the affected joint, limited range of motion (ROM), with the occurrence of the pelvic obliquity, windswept deformity, and scoliosis. In patients with severe cerebral palsy, hip displacement is often diagnosed late as a result of the silent nature of the displacement, communication difficulties, and the increased attention paid to other important issues such as feeding difficulties and seizure management. Identifying the subset of patients with an increased risk of hip displacement is essential in planning surveillance programs and early intervention (52).

Hip surveillance programs for children with CP has been adopted as a standard of care in several countries. Hip surveillance program is a targeted evaluation for children with CP with a regular schedule of physical examination and hip radiographs. Early identification and intervention have been shown to alter treatment outcomes, reduce the number of required reconstructive surgeries, and avoid the need of future salvage surgeries (56). Treatment options of hip subluxation or dislocation include hip adductor stretching, abduction bracing, botulinum toxin injections, soft tissue releases, and reconstructive bony procedures involving the proximal femur and/or acetabulum. This treatment aims to create a reduced, stable, mobile, and pain-free hip. Surgical management of hip subluxation or dislocation in children with CP is challenging knowing this condition often late detected (52).

### 3.7. Scoliosis

Scoliosis is a common deformity in non-ambulatory children with cerebral palsy (CP). This abnormal lateral curvature (Cobb angle > 10^o^) of the spine may be progressive and have negative effects on movement, posture, and everyday life (30). Neuromuscular scoliosis caused by altered neurological and muscle function is found in up to 15%−80% of CP patients. The variability of prevalence is due to the diversity of subjects' age, nature, the severity of neurological dysfunction, and radiography method (57). Scoliosis is often seen in younger ages in children with GMFCS V. The discovery of scoliosis in CP predates the Gross Motor Classification System (GMFCS), most studies agree that scoliosis is strongly linked with the level of global disability caused by CP (30, 58). A study conducted by Hagglund et al. (57) highlights the significant relationship between the GMFCS scale with the development of scoliosis, where 50% of patients categorized as GMFCS IV–V consistently develop greater Cobb angle curvature compared to 7% of children with GMFCS I–III (27 vs. 23^o^). A greater angle of scoliosis is associated with a higher risk of complications.

The greater degrees of scoliosis is associated with reduced quality of life through pain, hip and pelvic deformity, sitting and transfer difficulties, declining pulmonary function, and further motor dysfunction (57). Scoliosis in CP often leads to pelvic obliquity, which can increase the risk of windswept deformity, internal hip rotation, and reduced range of hip flexion on the elevated side, further development of these deformities may then cause hip subluxation or even dislocation (57, 58). Tissue injury and inflammation caused by hip subluxation and/or dislocation induce nociceptive pain in CP. An asymmetrical rib cage decreases the mobility and compliance of the ribs which will results in inefficient respiratory patterns, limits optimum respiratory volume, paradoxical breathing due to increased negative pressure in the upper thoracic cavity, and inefficient downward movement of the diaphragm (30).

Other studies focusing on CP agree that scoliosis is a combined result of spasticity, muscular imbalance around the spine, and poor muscle control that contribute to impaired trunk control (57, 58). Still unknown whether this is directly related to the primary cerebral injury or to secondary impairments, such as muscle weakness, spasticity, poor balance, or a non-ambulatory status (59).

Non-surgical management of scoliosis in children with CP is aiming to improve sitting control and reduce or modify curve progression. However, surgical management is necessary when the patient suffer from the complication of the scoliosis and aiming to achieve a balanced spine, prevention of curve progression and improvement in functional quality of life (58).

### 3.8. Sleep disorders and obstructive sleep apnea

Sleep plays a prominent role in children's physical growth, physiological, and neurological development (60). Sleep disorders reportedly occur more frequently in CP compared to typically developing children (23–46 vs. 20–30%), potentially affecting not only their quality of life but also their parents and family (61, 62). Children with CP commonly experience sleep disorders because of numerous negative consequences of the comorbid physical and medical conditions that often accompany CP, including restricted movement due to spasticity, pain, presence of epilepsy and use of antiepileptic medications, visual impairment, obstructive sleep apnea, and gastroesophageal reflux (63–65). Spasticity in these patients may lead to uncomfortable positioning and impair their ability to change position during sleep, resulting in persistent and increased pain associated with stiffness and joint contractures. Upper airway obstruction and gastroesophageal reflux can cause repeated arousal from sleep and induce sleep-related breathing disturbance (66). Despite the concerning prevalence of sleep disorders in CP, it is often overlooked in rehabilitation settings as clinicians rarely ask about the children's sleep habits during routine assessment (67).

Children with GMFCS level V are reportedly more likely to suffer from an abnormality of upper airway muscle tone, including laryngeal dystonia and severe laryngomalacia (68). Compared to healthy children, CP patients with obstructive sleep apnea (OSA) have upper airway narrowing which is caused by an anatomic factor (such as adenotonsillar hypertrophy) and abnormal muscle control. To compensate, there is an increased upper airway tone when the child is awake. However, during Rapid Eye Movement (REM) sleep, this compensatory mechanism is lost, leading to partial (hypopnea) or complete (apnea) airway closure (7). Children with severe neurologic impairment may have abnormal central control of respiration during sleep, which potentially leads to central apnea and/or periodic breathing. In addition, commonly used medications in children with cerebral palsy (antispastic, antiepileptic) can also depress upper airway maintenance musculature (69).

A detailed sleep history assessment and polysomnography as a gold standard examination is essential in establishing a diagnosis (69). Loud snoring, difficulty breathing during sleep, and apnea witnessed by parents are the three most predictive symptoms of OSA (70). The presence of OSA significantly reduced the child's and parents' quality of life, failure to thrive, cardiorespiratory compromise, neurobehavioral issues, non-reversible deficits, and eventually death (71).

Most of the OSA case often requires surgical procedure. Tonsillectomy and adenoidectomy, or uvulapalatopharyngoplasty can be employed successfully to treat OSA in children with CP and will include tongue base suspension in severe cases, however anatomical structure and severity of the obstruction must also be considered (72).

### 3.9. Pain

Pain is common in spastic cerebral palsy children, with a reported prevalence of 65%−78%, and is a major influence following subjective wellbeing and leading to reduced participation in everyday life (73). Johnson and her colleagues (74) stated that CP children with spasticity are most often presented with chronic nociceptive musculoskeletal pain which develops from hip dislocation. A systematic review by Ostojic (75) identified that pain in CP children, apart from being from several sources, including hip dislocation/subluxation, dystonia, and musculoskeletal deformity, also emphasizes pain from interventions used in CP management. With spasticity affecting all their limbs, these children have problems expressing their experience toward pain, through lower pain behavior frequency (76).

Most complaints of pain in spastic CP children are in the form of chronic secondary pain of musculoskeletal origin; it is often caused by joint misalignment, muscle spasms, and osteoporosis, while the primary source of pain from complex regional pain syndrome remains rare (73). Pain from muscle spasms is caused by muscle contractions that lead to vascular compression. A compressed blood vessel creates an ischemic condition, while muscle spasms demand large amounts of oxygen, resulting in the activation of nociceptors (pain receptors of noxious stimuli). The pain itself may also cause muscle spasms in the affected point, creating a vicious cycle (73, 77). Other causes of muscle spasms are fatigue, inflammation, and overuse. Spasticity combined with muscle weakness and soft tissue changes as seen in spastic CP children will eventually contribute to secondary painful conditions, such as contractures. This will lead to joint deformities and misalignment, especially in weight-bearing joints. Osteoporosis occurs at an early age in spastic CP children due to decreased mechanical loading. Non-ambulant children have higher risks of painful pathological fractures, especially in the lower extremities, that are not only painful but also prolong immobilization (73, 78). Postural asymmetries are also associated with pain. Back pain is more frequent with scoliosis especially in individuals with quadriplegic CP (65%−74%) since their neurologic handicaps affect their ability to maintain posture, thus their ribs impinge against the elevated side of the pelvis (77).

While not as prevalent as chronic musculoskeletal pain, other origins of pain can also occur in spastic quadriplegic children with CP. Visceral pain covers physiological mechanisms that cause abdominal pain in children with CP, where disturbances of neural modulation of colonic motility play a role in cases of constipation and GERD (48). Nevertheless, visceral pain and its correlation with spastic CP has not been fully understood (73). Bruxism is another risk factor for temporomandibular disorders that give rise to symptoms like jaw pain and secondary headaches (79). Acute pain rises from interventions done to the underlying disease that accompanies spastic CP children, such as surgery, botulinum neurotoxin A injections, and physiotherapy. Therefore, they are likely to activate nociceptors. Acute nociceptive pain occurs after surgical procedures by increasing muscle spasms toward post-operative scars (73, 74). Though, physiotherapy is essential for the improvement of motor-related functions, balance, and posture; 45% of children had pain provoked during physiotherapy. The use of standing frames during physiotherapy are reported to be painful for 14% of children (73).

Although both paracetamol or ibuprofen are highly used as a medication for acute or chronic pain in children with CP, there are also non-pharmacology medication to treat acute pain such as rest, massage, distraction, comfort, and stretching/exercising. Meanwhile, children with chronic pain more likely to engaged with massage, thermotherapy, and hydrotherapy (75).

### 3.10. Secondary sensory processing problems

Inadequate postural adjustments and abnormal movement patterns used by the child with CP affect sensory experiences in three ways. First, the repetition of inadequate postural adjustments and abnormal movement patterns influences the proprioceptive-kinesthetic feedback received by the child. Second, the vestibular and visual experiences are limited due to the lack of movement. Third, tactile experiences are restricted because the child receives limited tactile input (80, 81). The development of the body scheme depends on adequate processing of proprioceptive, tactile, and vestibular input (81). Vestibular processing deficits generate balance impairment with poor muscle control, Barakat MKA and his colleagues (82) promote that the reason might be due to diminishing thalamocortical projections from the thalamus to the primary somatosensory cortex (S_1_), which defects somatosensory processing in spastic quadriplegic CP children (83).

Sensory feedback provided by abnormal movement patterns is integrated into the body scheme, abnormal muscle tone, inadequate postural adjustments, abnormal movement patterns, asymmetries, and inefficient weight-bearing and weight-shifting patterns interfere with the child's reception of sensory input and affect the development of body scheme, the integration of the two sides of the body, the crossing of the midline, and motor planning, which hinder functional performance (80). Primary sensory processing problems of the child with cerebral palsy also interfere with the child's performance (69).

The child with CP may exhibit three types of sensory processing deficits: modulation deficits, discrimination deficits, and hyporesponsiveness to sensory input or registration deficits (81, 84). Modulation deficits are evidenced in the child who does not regulate sensory input and either overreacts or underreacts to it. These children may often exhibit fluctuating arousal levels that may depend on environmental factors. Children with sensory modulation deficits are also often described as children whose response to sensory input varies from one extreme to the other. This variation occurs within the same session or between sessions. Modulation disorders are due to the lack of inhibition of extraneous sensory input occurring at several levels of the central nervous system, including the cortex (81). Registration and modulation deficits impact the level of arousal and attention, therefore, hindering learning and performing purposeful tasks (84). The child who tends to overreact to input or who is often defensive may exhibit increased arousal levels and may have difficulty attending.

Children with spastic quadriplegic CP may also present a maintained hyporesponsivity to sensory experiences. Hyporesponsiveness to sensory input is evident by a delayed or diminished response or failure to respond to sensory input. Children who do not register or who are hyporesponsive often exhibit a decreased arousal level and have difficulty maintaining their attention on a task (83).

Sensory Integration Therapy (SIT) aims to provide CP child with graded sensory experiences. SIT is a clinical-based technique that emphasizes the therapist-child interaction and employs play-based sensory and motor exercises to promote sensation processing integration (85). Furthermore, SIT intervention had a significant effect on improving gross motor function in the children with CP (86).

A summary of multisystem consequences in spastic quadriplegic CP children can be seen in Table 1.

**Table 1:** Summary of multisystem consequences in spastic quadriplegic CP children.

**Systems**	**Conditions**	**Causes**	**Consequences**
Gastrointestinal	Dysphagia	•Oral motor disorders • Pharyngoesophageal dyskinesia • Intestinal dysmotility • Poor feeding posture	•Aspiration pneumonia • Feeding difficulties • Inadequate nutritional status, poor child's growth
	GERD	•Immobilization • Scoliosis • Delayed gastric emptying • Inadequate oral intake • Abnormal autonomic control of gastrointestinal motility • Medications (anti-epileptic and anti-spastic drugs)	•Dysphagia • Dental erosions • Feeding difficulties • Poor weight gain • Recurrent vomiting • Aspirations
	Drooling	•Oromotor control dysfunction • Poor head and neck control (forward leaning posture) • Medications (anti-epileptic drugs)	•Chapped skin, dehydration • Dental occlusion • Poor oral hygiene • Chewing disorders • Loss of self-confidence, self-isolation • Coughing, choking, vomiting • Pneumonia aspiration
	Constipation	•Poor gastrointestinal motility • Lack of fluid, dietary fibers • Medications (BoMT-A, anticonvulsants, anticholinergics)	•Pain
Respiratory	Impaired respiratory function	•Respiratory muscle paralysis • Inefficient biomechanics of breathing structures • Limited chest expansion • Immobility • Gastrointestinal (dysphagia, GERD)	•Aspiration pneumonia • Atelectasis, bronchiectasis • Chronic obstructive and restrictive lung diseases
Musculoskeletal	Hip subluxation and dislocation	•Spasticity • Muscle weakness	•Hip instability • Pelvic obliquity • Windswept deformity • Scoliosis
	Scoliosis	•Spasticity • Muscular imbalance • Poor muscle and trunk control	•Pain • Hip and pelvic deformity • Sitting and transfer difficulty • Declining pulmonary function
Sleep	Sleep disorder and OSA	•Spasticity • Pain • GERD • Medications (anti-epileptic and anti-spastic drugs) • Epilepsy • Visual impairment • Abnormal muscle control (laryngeal dystonia, laryngomalacia)	•Persistent and increased pain associated with stiffness and joint contractures • Partial (hypopnea) or complete (apnea) airway closure • Failure to thrive • Cardiorespiratory compromise • Neurobehavioral issues • Reduced quality of life
Pain	Pain	•Joint misalignment, deformity, and contractures • Muscle spasm • Osteoporosis • Postural asymmetries • Gastrointestinal (GERD and constipation)	•Reduced quality of life and participation • Inducing stress for patients and family/caregiver
Sensory processing	Secondary sensory processing disorder	•Inadequate postural adjustments • Abnormal movement patterns • Limited visual, vestibular, and tactile experiences	•Poor reception of sensory input • Interfere integration of both sides of the body, development of body scheme, and motor planning • Poor functional performance • Difficulty maintaining attention on a task

## 4. Management strategy in spastic quadriplegic CP children

Children with spastic quadriplegic CP have numerous multisystem consequences that require attentions and best managed based on standard guidelines (87). Deciding the goals and benefits of the treatments are the essential steps to begin the treatments. The risks and benefits of any particular intervention should be carefully considered. Goals therapy of CP spastic quadriplegic children are aimed to reduce pain, prevent or decrease contractures, improve ambulation, facilitate activities of daily living (ADL), promote participation in rehabilitation, improve the ease of care and safety (88). A multidisciplinary team approach provides the best model for medical care of children with CP across their lifespan to manage various associated and secondary conditions as well as address support system and psychosocial needs (88).

## 5. Conclusion

Healthcare professionals who treat children with cerebral palsy spastic quadriplegic notice that many things get worse, not better. Understanding the development of multisystem compensations and the consequences of atypical movement patterns is extremely important. It is true that central nervous system (CNS) lesion in CP is non-progressive, however the multisystem consequences may impair overall function over time. Considering multisystem compensations and consequences that occur in spastic quadriplegic CP children, designing goals therapy and forming an interdisciplinary team to manage various secondary conditions is essential. An understanding of how multisystem compensations and consequences develop offers the opportunity to intervene as much as possible before they begin.

## Author contributions

LW was responsible for the literature review, conceptual design, manuscript preparation, and editing process of this article.
